# The Protective Effect of Astaxanthin on Cognitive Function via Inhibition of Oxidative Stress and Inflammation in the Brains of Chronic T2DM Rats

**DOI:** 10.3389/fphar.2018.00748

**Published:** 2018-07-10

**Authors:** Yonghao Feng, Aiqun Chu, Qiong Luo, Men Wu, Xiaohong Shi, Yinghui Chen

**Affiliations:** ^1^Department of Endocrinology, Jinshan Hospital, Fudan University, Shanghai, China; ^2^Department of General Medicine, Shihua Community Health Service Center, Shanghai, China; ^3^Department of Neurology, Huashan Hospital North, Fudan University, Shanghai, China

**Keywords:** astaxanthin, type 2 diabetes mellitus, oxidative stress, inflammatory response, Nrf2, cytokines

## Abstract

Currently, there are no effective treatments for diabetes-related cognitive dysfunction. Astaxanthin (AST), the most powerful antioxidant in nature, exhibits diverse biological functions. In this study, we tried to explore whether AST would ameliorate cognitive dysfunction in chronic type 2 diabetes mellitus (T2DM) rats. The T2DM rat model was induced via intraperitoneal injection of streptozotocin. Forty Wistar rats were divided into a normal control group, an acute T2DM group, a chronic T2DM group, and an AST group (treated with AST at a dose of 25 mg/kg three times a week). The Morris water maze test showed that the percentage of time spent in the target quadrant of the AST group was identical to that of the chronic T2DM group, while the escape latency of the AST group was decreased in comparison to that of the chronic T2DM group. Histology of the hippocampus revealed that AST ameliorated the impairment in the neurons of diabetic rats. Western blot showed that AST could upregulate nuclear factor erythroid 2-related factor 2 (Nrf2) and heme oxygenase 1 (HO-1) expression and inhibit nuclear transcription factor kappa B (NF-κB) p65 activation in the hippocampus. We found that AST increased the level of superoxide dismutase (SOD) and decreased the level of malondialdehyde (MDA) in the hippocampus. In addition, the levels of interleukin 1 beta (IL-1β) and interleukin 6 (IL-6) were reduced in the AST group compared with those in the chronic T2DM group. The findings of this research imply that AST might inhibit oxidative stress and inflammatory responses by activating the Nrf2-ARE signaling pathway.

## Introduction

Diabetes mellitus (DM) has become the most common chronic metabolic disease that threatens global health. The most common form of DM is type 2 diabetes mellitus (T2DM), characterized by insulin resistance and relative insulin insufficiency (Chatterjee et al., 2017). T2DM is associated with long-term complications that affect the eyes, kidneys, heart, blood vessels, and brain. Increasing evidence has indicated that patients with chronic T2DM can exhibit various intracranial neuropathies and neurobehavioral changes (McCrimmon et al., 2012). Lately, the incidence of diabetes-related cognitive dysfunction has increased with the aging of the global population. This diabetes-related cognitive dysfunction is connected to the duration of diabetes and to the level of blood glucose; it can also be partially prevented by strict blood glucose control (Munshi et al., 2006).

The multifactorial process of cognitive dysfunction in DM is not yet completely understood (Gaspar et al., 2016), however, classical factors, such as oxidative stress and inflammatory responses, may contribute to the pathogenesis of diabetes-related cognitive dysfunction, leading to abnormal brain structure and dysfunction in diabetic rats (Mastrocola et al., 2005; Wang et al., 2015a). Nuclear factor erythroid 2-related factor 2 (Nrf2) plays an anti-oxidative role by binding with the antioxidant responsive element (ARE) in the nucleus (Nguyen et al., 2009). In addition, Nrf2 signaling is involved in inflammatory responses (Ahmed et al., 2017).

Astaxanthin (AST) is the most potent antioxidant found in nature (Ambati et al., 2014). It is one of the carotenoids, and the hydroxyl and keto endings located on ionone ring at both ends. Due to the optical rotation of hydroxyl at both ends, AST mainly has 3 isomers of 3S-3′S, 3R-3′S, and 3R-3′R (Guerin et al., 2003). Among them, the 3S-3′S has stronger antioxidant properties than ordinary carotenoids, which makes it easily penetrate the cell membrane and maintain the integrity of the membrane structure. The unique chemical structure of AST enables it to cross the blood-brain barrier easily and plays an important role in the treatment of central nervous system diseases (Zhang et al., 2014b; Ying et al., 2015). AST is capable of various biological characteristics, such as anti-inflammation, anti-oxidation, anti-apoptosis, and other biological characteristics (Zhang et al., 2014a,b; Ying et al., 2015). It has been demonstrated that AST can inhibit oxidative stress in human vascular endothelial cells exposed to glucose fluctuations (Regnier et al., 2015). Meanwhile, AST is considered able to alleviate cognitive dysfunction in diabetic mice by inhibiting inflammatory responses (Zhou et al., 2015). Whether AST inhibits oxidative stress and inflammatory responses in T2DM rats with cognitive dysfunction via Nrf2/ARE signaling is unclear. To investigate the therapeutic effect and potential mechanism of AST in chronic T2DM rats with cognitive dysfunction, we performed this study.

## Materials and Methods

### Reagents

Astaxanthin (purity ≥ 98%) was purchased from Shanghai Kaimaishu Biotechnology Co., Ltd. Streptozocin (STZ) was purchased from Sigma company. Chloral hydrate was purchased from Shanghai Engineering Show Biological Technology Co., Ltd. The MILLIPLEX MAP Rat Cytokine/Chemokine Magnetic Bead Panel was purchased from Merck Millipore.

### Experimental Grouping and Establishment of the T2DM Rat Model

**FIGURE 1:**
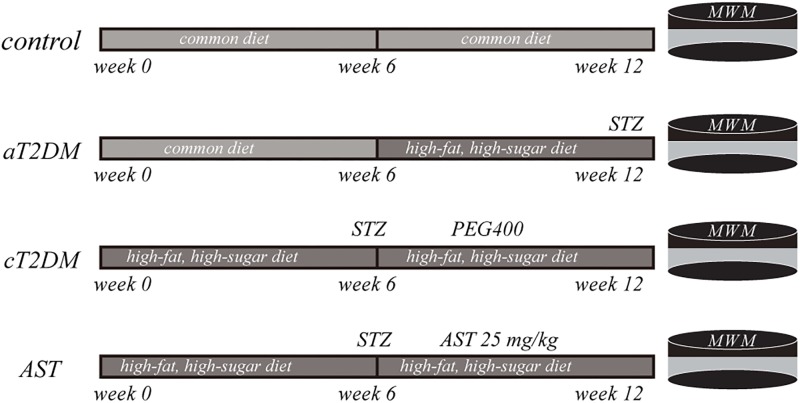
A schematic flow chart exhibiting the experimental paradigm of the present study. aT2DM, acute type 2 diabetes mellitus; cT2DM, chronic type 2 diabetes mellitus; AST, astaxanthin. WMM: Morris water maze; STZ: streptozocin; PEG400: polyethylene glycol 400.

A total of 40 male Wistar rats (140–160 g) were purchased from Shanghai Slack Laboratory Animal Co., Ltd. These rats were utilized in conformity with the regulations of the Shanghai Animal Management Commission. The experimental procedures were approved by Ethics Committee of Jinshan Hospital, Fudan University, and the authorization number was Jin Medical Ethics 2016(21). These animals were kept under a 12 h light/dark cycle at 20 ± 2°C and a relative humidity of 60%. The rats were divided into a control group, an acute T2DM (aT2DM) group, a chronic T2DM (cT2DM) group, and an AST group, with 12 rats in each group. The control group was fed a common diet for 12 weeks. The aT2DM group was fed a common diet for 6 weeks and then was given a high-fat, high-sugar diet containing 10% lard, 20% sucrose, and 70% regular feed for another 6 weeks. Then, the rats were injected with STZ intraperitoneally at a dose of 35 mg/kg. Fasting blood glucose levels (fasted for 12 h, with water) were determined 72 h later. The T2DM rats were considered successfully established with blood glucose levels >16.7 mmol/L. The cT2DM group and the AST group were directly fed a high-fat, high-sugar diet for 6 weeks. After, the T2DM rats were produced in the same manner. AST, dissolved in polyethylene glycol 400 (PEG400) (Choi et al., 2011), was administered intraperitoneally at a dose of 25 mg/kg (Tripathi and Jena, 2010), three times a week for 6 weeks. The cT2DM group was injected intraperitoneally with the same amount of PEG400. During this period, the two groups of rats were still fed a high-fat, high-sugar diet (**Figure 1**).

### Blood Glucose Measurement

Abbott blood glucose meter and test strips were used to measure fasting blood glucose in rats. At first, the test strip was inserted into the groove in the Abbott blood glucose meter. Then about 0.05 mL blood samples of the rats were taken from the tail vein 72 h after the intraperitoneal injections. At last, the obtained venous blood was transferred to the detection area of test strip. Before the rats were sacrificed, the FBG level of each rat was measured again in the same way.

### Morris Water Maze Test

The Morris water maze test is used to detect spatial learning and memory in rodents that rely on distal cues to navigate from start locations around the perimeter of an open swimming tank to locate a submerged platform. Spatial learning is evaluated via repeated place navigation, and reference memory is assessed by a preference for the platform area when the platform is absent. The spatial learning and memory of the rats were assessed using the Morris water maze test system (Shanghai Xin Soft Information Technology Co., Ltd.). The place navigation test was performed four times a day for 4 consecutive days with intervals of 15–20 min between each test, and a probe trial was conducted on the fifth day. In each test, a rat was released into the water from each quadrant, in turn, facing the pool wall. The rats were allowed to swim for a maximum of 60 s until they found the platform. If the rat failed to find the platform in 60 s, it was assigned a score of 60 s and was gently guided to the platform and allowed to stay on it for 15 s. On the probe trial day, the platform was removed, and each rat was released into the pool from the position opposite the target quadrant. The swimming paths of the rats were recorded for 60 s. The room was maintained at 20 ± 2°C, and the water in the pool was changed every day.

### Histology Analysis

The four groups of rats were deeply anesthetized via intraperitoneal injections with 10% chloral hydrate at a dose of 30–35 mg/kg. The rats were first perfused with 100 mL of physiological saline in the left ventricle and then with 100 mL of 4% paraformaldehyde in 0.1 M phosphate buffer (pH 7.4) at 4°C. Brain tissue was obtained and dissected into four small blocks. After the brain blocks were soaked in 4% paraformaldehyde for 24 h, they were dehydrated and embedded in paraffin. Coronal sections (5 μm thick) were stained with hematoxylin-eosin (H-E). The stained sections were then viewed and photographed with an Olympus BX53 light microscope (Tokyo, Japan).

### Western Blot Assay

After the rats were deeply anesthetized, they were sacrificed to directly obtain hippocampal tissue. Total protein was obtained from the hippocampal tissue with sodium dodecyl sulfate lysis buffer (Beyotime, Shanghai, China) and mixed with 1 mM phenylmethylsulfonyl fluoride (Beyotime). Equal amounts of protein were examined with 10% SDS-polyacrylamide gel electrophoresis and then transferred to a polyvinylidene difluoride membrane (Merck Millipore, Billerica, MA, United States). The membrane was blocked with 5% bovine serum albumin at room temperature for 1 h and then incubated with primary antibodies at 4°C for 12 h. The primary antibodies were Nrf2 antibody (1:500 dilution; Santa Cruz Biotechnology, Santa Cruz, CA, United States), HO-1 (1:500 dilution; Abcam, Cambridge, MA, United States), NF-κB p65 antibody (1: 1,000 dilution; Cell Signaling Technology, Danvers, MA, United States), anti-tubulin and anti-β-actin primary antibody (1:5,000 dilution; Proteintech Group Inc., Chicago, IL, United States). After washing, suitable horseradish peroxidase-conjugated secondary antibodies (1: 5,000 dilution) were incubated with the membranes at room temperature for at least 2 h. The bound antibody was visualized using enhanced chemiluminescence solution (Merck Millipore), and the signals were detected using ECL-Plus (Merck Millipore). The relative density of each group was quantified using the ImageJ Software (National Institutes of Health, Bethesda, MD, United States).

### Determination of MDA and SOD

The determination of superoxide dismutase (SOD) and malondialdehyde (MDA) in the hippocampal tissue was quantified using a SOD kit (Fujian Fuyuan Biological Technology Co., Ltd., Fujian, China) and an MDA kit (Nanjing Jiancheng Bioengineering Institute, Nanjing, China). A 25 mg sample of hippocampal tissue was weighed and added to 475 μl of physiological saline. The mixture was homogenized using a vortex mixer. Supernatants were obtained after centrifugation at 4,000 RPM for 5 min. The SOD was determined by pyrogallol autoxidation method. Reagent 1 and reagent 2 in the SOD reagent kit are formulated in a ratio of 5:1. Then 2 mL of working solution and 100 ul of sample were added to each detection well and were mixed. Then the mixed liquor was detected by automatic biochemical analyzer at a wavelength of 420 nm and the temperature of 37°C. At last the SOD level of each sample were calculated by automatic biochemical analyzer. The level of MDA was quantified by thiobarbituric acid (TBA) method. In the process of measuring, standard tube, standard blank tube, and measuring tube were used. The 4 mL of working solution was added to the three kinds of tube respectively. Besides, 100 μL of standard sample was added to standard tube. At the same time, 100 μL of anhydrous alcohol was added to standard blank tube and 100 μL of sample was added to measuring tube. The mixed solution of each tube was homogenized using a vortex mixer, and then were incubated for 80 min at 95° water temperature. When they were cool down by running water and then were centrifuged at 3,500 RPM for 10 min. The 200 μL of supernatants were obtained from each tube. The absorbance of each sample was measured using a microplate reader at a wavelength of 532 nm. Each sample was calculated according to the conversion formula.

### Determination of Inflammatory Cytokines

When the rats were deeply anesthetized, the blood was collected from the heart. The blood samples were centrifuged at 4,000 RPM for 5 min, and then plasma samples were obtained. The plasma samples were stored at -80°C when not in use. The levels of interleukin 1 beta (IL-1β) and interleukin 6 (IL-6) in the serum were quantified using the MILLIPLEX MAP Rat Cytokine/Chemokine Magnetic Bead Panel as per the manufacturer’s instructions (Merck Millipore, Billerica, MA, United States). The IL-6 and IL-1β cytokines were determined. Premixed magnetic beads conjugated to specific antibodies for 12 analytes were mixed with 25 μL of plasma samples in 96-well plates. Plates were preserved to avoid light and were incubated on a shaker at 4°C for less than 12 h, and then magnetic beads were washed for three times with 200 μL of wash buffer. Then detection antibodies were added to each well, and the mixtures were incubated at room temperature for 1 h. Streptavidin-phycoerythrin was added to each well, and the mixtures were incubated at room temperature for 30 min. The magnetic beads were resuspended in sheath fluid, and plates were assayed on a Luminex^®^ 200^TM^ system with xPONENT^®^ software. The experimental data are presented in pg/mL as a unit.

### Statistical Analysis

All the measurement data are expressed as the mean ± standard deviation (SD). The SPSS 23.0 (IBM Corp., Armonk, NY, United States) statistical software was used for the analyses. Two sets of data collected from the same sample at different times were analyzed using paired *t*-tests. One-way ANOVA was used to compare the differences among multiple samples. Repeated-measures ANOVA was used to analyze continual measurement data for the same subject among multiple samples. Pearson correlation analyses were carried out to clarify the relationship between Nrf2 and SOD and between MDA and inflammatory cytokines. Differences were statistically significant at *P <* 0.05.

## Results

### Effect of AST on the Blood Glucose Levels

As shown in **Table 1**, the fasting blood glucose level of each group was different at the 6th week (*F* = 941.6, *P <* 0.01). The blood glucose levels of the cT2DM group and of the AST group were significantly higher than that of the control group at the 6th week (*P <* 0.01). There was no significant difference between the AST group and the cT2DM group (*P* = 0.785, *P* > 0.05). The fasting blood glucose levels of the control, cT2DM, and AST groups were not different at the 12th week (*F* = 0.388, *P* > 0.05). The blood glucose level of the AST group was slightly lower than that before AST treatment, but there was no significant difference between the two groups (*t* = 0.464, *P* > 0.05). This finding implied that AST had no obvious effect on the blood glucose levels of the diabetic rats in this study.

**Table 1:** Effect of AST on the blood glucose level in each group (unit: mmol/l).

Time	Control	aT2DM	cT2DM	AST
6th week	2.45 ± 0.59	2.97 ± 0.63	19.21 ± 1.20^∗∗^	19.09 ± 1.28^∗∗^
12th week	2.82 ± 0.35	18.58 ± 1.32	18.27 ± 1.13	18.09 ± 1.29


*Data are shown as the mean ± SD. ^∗∗^P < 0.01. aT2DM, acute type 2 diabetes mellitus; cT2DM, chronic type 2 diabetes mellitus; AST, astaxanthin.*

### Effects of AST on Cognitive Dysfunction

**FIGURE 2:**
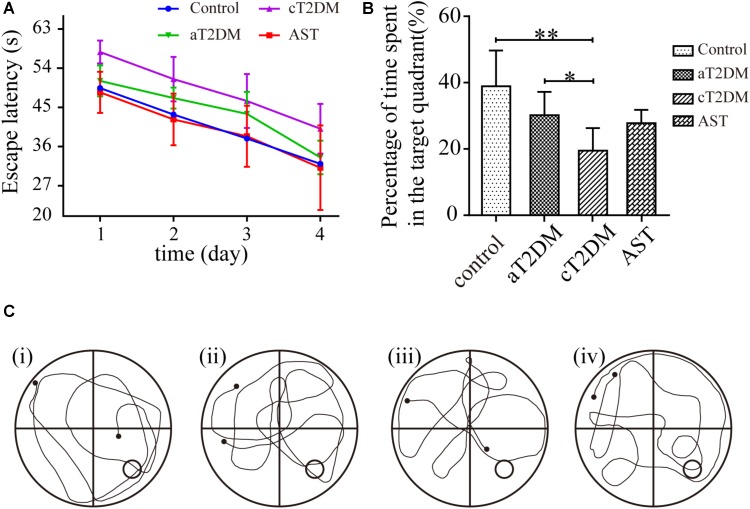
Effect of AST on the spatial learning and memory of each group. This figure exhibits the cognitive function (as measured by the Morris water maze test) of the control, aT2DM, cT2DM, and AST groups. The line chart shows the average escape latency **(A)** in the place navigation test over 4 consecutive days. In the probe trial, the percentage of time spent in the target quadrant **(B)**, and the typical swimming traces **(C)** were recorded. (i) control group; (ii) aT2DM group; (iii) cT2DM group; (iv) AST group. Data are shown as the mean ± SD. ^∗^*P <* 0.05, ^∗∗^*P <* 0.01. aT2DM, acute type 2 diabetes mellitus; cT2DM, chronic type 2 diabetes mellitus; AST, astaxanthin.

It has been considered that cognitive function is correlated with hippocampal bioenergetics. Thus, we performed the place navigation and probe trials to explore whether AST would successfully preserve the cognitive dysfunction of diabetic rats. Repeated-measures ANOVA proved that the escape latencies were significantly different between each group (*F* = 5.893, *P <* 0.01, **Figure 2A**) and among the different periods (*F* = 55.568, *P <* 0.01, **Figure 2A**). Compared with the control group, the escape latency in the aT2DM group was unchanged (*P* = 0.201, *P* > 0.05), while the escape latency in the cT2DM group was increased (*P* = 0.002, *P <* 0.01). The escape latency in the AST group was shorter than that in the cT2DM group after the treatment with AST (*P* = 0.001, *P <* 0.01). In the probe trial session, the percentage of time spent in the target quadrant was different for each group (*F* = 6.706, *P <* 0.01). The percentage of time spent in the target quadrant was not significantly different between the control group and the aT2DM group (*P* = 0.058, *P* > 0.05). The percentage of time spent in the target quadrant in the cT2DM group was less than that of the control group (*P* < 0.01). Under treatment with AST, the percentage of time spent in the target quadrant in the AST group was identical to that in the cT2DM group (*P* = 0.072, *P <* 0.05). The behavioral data indicated that the cT2DM group rats displayed severe cognitive dysfunction in terms of both damaged spatial learning (**Figure 2A**) and spatial memory (**Figure 2B**). In addition, there existed no serious cognitive impairments in the early stages of diabetes. Under the treatment with AST, the spatial learning of the diabetic rats was evidently improved (**Figure 2A**). However, the spatial memory deficits were not obviously alleviated (**Figures 2B,C**). The data acquired from the Morris water maze test revealed that AST could ameliorate the cognitive impairment of chronic diabetic rats to a certain extent.

### Histological Examination

**FIGURE 3:**
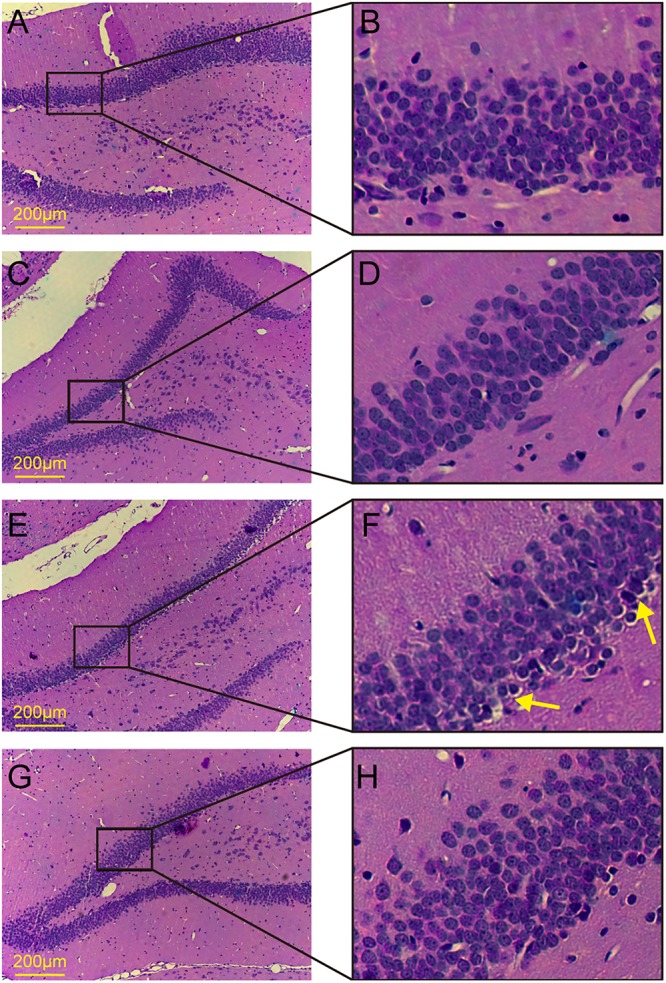
HE staining of the dentate gyrus in the hippocampus. **(A,B)** Control group; **(C,D)** acute type 2 diabetes group; **(E,F)** chronic type 2 diabetes group; **(G,H)** astaxanthin group.

The dentate gyrus has been considered to be closely related to cognitive function. To observe the morphological changes in the dentate gyrus, we performed HE staining. No obvious histological changes were observed among the control group, the aT2DM group, or the AST group (**Figures 3A–D,G,H**). Meanwhile, karyopyknosis was observed in the dentate gyrus of the cT2DM group hippocampus compared with those of the control group and the AST group (**Figures 3E,F**). This finding indicated that the morphological impairment was clearly distinguished in chronic diabetic rats, and AST could ameliorate this morphological impairment.

### The Expression Levels of Nrf2, HO-1, SOD, and MDA in Each Group

**FIGURE 4:**
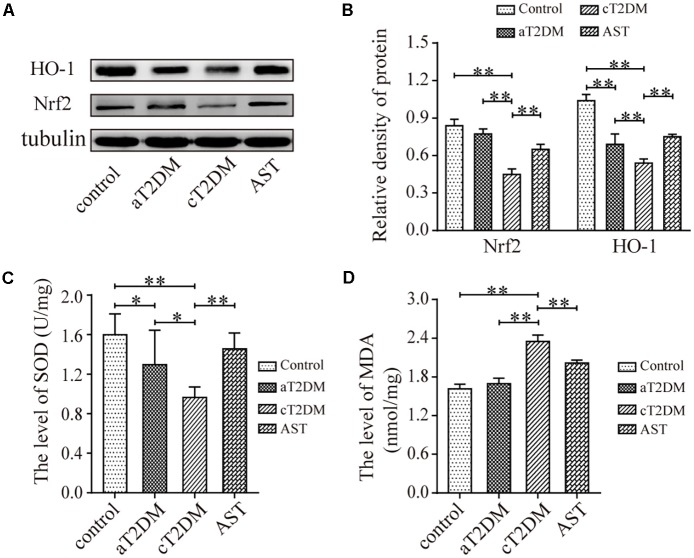
The expression levels of Nrf2, HO-1, SOD, and MDA in each group. Representative protein bands of Nrf2 and HO-1 **(A)**; Nrf2/tubulin and HO-1/tubulin ratio according to band density **(B)**; The average level of SOD in each group (**C**, units: U/mg); The average level of MDA in each group (**D**, units: nmol/mg). Data are shown as the mean ± SD. ^∗^*P <* 0.05, ^∗∗^*P <* 0.01. aT2DM, acute type 2 diabetes mellitus; cT2DM, chronic type 2 diabetes mellitus; AST, astaxanthin.

Western blot (**Figures 4A,B**) analysis showed that the expression levels of Nrf2 were significantly different among the groups (*F* = 44.858, *P <* 0.01). Compared with the control group, the expression of Nrf2 in the aT2DM group was statistically unvaried (*P* = 0.109, *P* > 0.05), while the expression of Nrf2 in the cT2DM group was significantly reduced (*P* = 0.003, *P <* 0.01). Meanwhile, the expression of Nrf2 in the cT2DM group was decreased compared with that in the aT2DM group (*P* < 0.01). The expression of Nrf2 in the AST group was higher than that in the cT2DM group (*P* < 0.01). The expression levels of HO-1 were also significantly different between each group (*F* = 46.616, *P <* 0.01) (**Figures 4A,B**). The expression of HO-1 in the aT2DM group was lower than that in the control group (*P* < 0.01) but significantly higher than that in the cT2DM group (*P* = 0.008, *P <* 0.01). The expression of HO-1 in the cT2DM group was visibly increased in comparison to that in the control group (*P* < 0.01). The expression of HO-1 in the AST group was higher than that in the cT2DM group (*P* = 0.001, *P <* 0.01). This finding revealed that AST could upregulate the expression of Nrf2 and HO-1 and that Nrf2 and HO-1 were slightly changed in the early periods of diabetes. The levels of SOD were significantly different in each group (*F* = 7.496, *P <* 0.01, **Figure 4C**). Compared with the control group, the level of SOD in the aT2DM group was slightly decreased (*P* = 0.044, *P <* 0.05) but was higher than that in the cT2DM group (*P* = 0.033, *P <* 0.05). The level of SOD in the cT2DM group was decreased in comparison to that in the control group (*P* < 0.01). The level of SOD in the AST group was higher than that in the cT2DM group under treatment with AST (*P* = 0.003, *P <* 0.01). This result revealed that the ability to resist oxidative stress in the early stages of diabetes was diminished and that AST elevated the ability to resist oxidative stress in the diabetic rats. The levels of MDA were significantly different between each group (*F* = 91.727, *P* < 0.01, **Figure 4D**). Compared to the control group, the level of MDA in the aT2DM group was unchanged (*P* = 0.113, *P* > 0.05), while the level of MDA in the cT2DM group was increased (*P* < 0.01). Meanwhile, the level of MDA in the cT2DM group was higher than that in the aT2DM group (*P* < 0.01). The level of MDA in the AST group was lower than that in the cT2DM group after treatment with AST (*P* < 0.01). This finding demonstrated that AST could clearly inhibit lipid peroxidation and that there was no obvious lipid peroxidation in early periods of diabetes. According to the above results, it was implied that the oxidation and anti-oxidation systems are slightly unbalanced in the early stage of diabetes and that AST inhibits the level of oxidative stress.

### The Expression Levels of NF-κB p65 and Inflammatory Cytokines in Each Group

**FIGURE 5:**
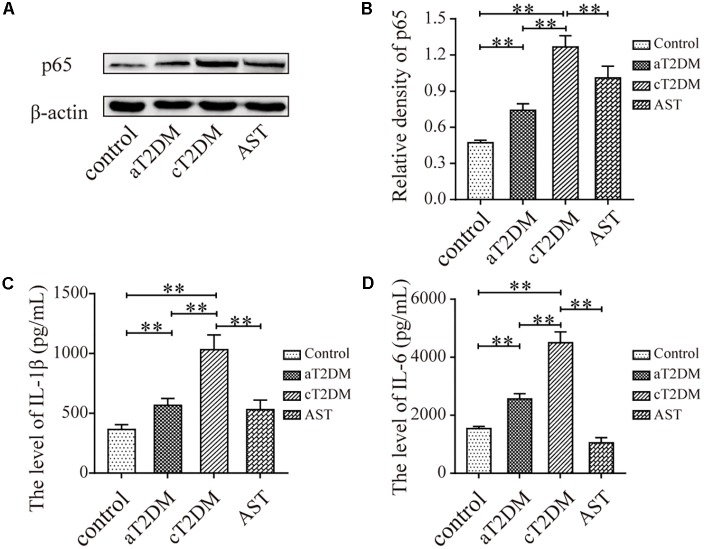
The expression levels of NF-kB p65, IL-1β, and IL-6 in each group. Immunoblots **(A)** and histogram **(B)** showing the expression of NF-kB p65. The levels of IL-6 **(C)** and IL-1β **(D)** are also displayed in the histogram. Data are shown as the mean ± SD. ^∗∗^*P* < 0.01. aT2DM, acute type 2 diabetes mellitus; cT2DM, chronic type 2 diabetes mellitus; AST, astaxanthin.

The immunoblots (**Figures 5A,B**) indicated that the expression levels of NF-κB p65 were significantly different between each group (*F* = 64.459, *P <* 0.01). Compared with the control group, the expression levels of NF-κB p65 in the aT2DM and cT2DM groups were increased (*P* < 0.01). However, the expression of NF-κB p65 in the aT2DM group was lower than that in the cT2DM group (*P* < 0.01). The expression level of NF-κB p65 in the AST group was significantly lower than that in the cT2DM group (*P* = 0.003, *P <* 0.01). This result indicated that AST could down-regulate the expression of NF-κB p65 to a certain extent and that NF-κB p65 was wildly activated in the early periods of diabetes. The results of the multiplex immunoassay (**Figures 5C,D**) showed that the expression levels of IL-β and IL-6 in each group were significantly different (*F* = 62.072, *P <* 0.01; *F* = 218.975, *P <* 0.01). The expression of IL-1β in the aT2DM group was significantly higher than that in the control group (*P* < 0.01) but lower than that in the cT2DM group (*P* < 0.01). Meanwhile, the expression of IL-1β in the cT2DM group was increased in comparison to the control group (*P* < 0.01). The expression of IL-1β in the AST group was lower than that in the cT2DM group (*P* < 0.01). Compared with the control group, the expression of IL-6 in the aT2DM group was significantly increased (*P* < 0.01) but was lower than that in the cT2DM group (*P* < 0.01). The expression level of IL-6 in the cT2DM group was increased in comparison to the control group (*P* < 0.01). The expression level of IL-6 in the AST group was lower than that in the cT2DM group (*P* < 0.01). This finding indicated that a mild inflammatory reaction arose in the early stages of diabetes and that AST could down-regulate the expression of inflammatory cytokines in chronic diabetic rats.

### The Correlation Between Nrf2 and SOD, MDA, and Inflammatory Cytokines

**FIGURE 6:**
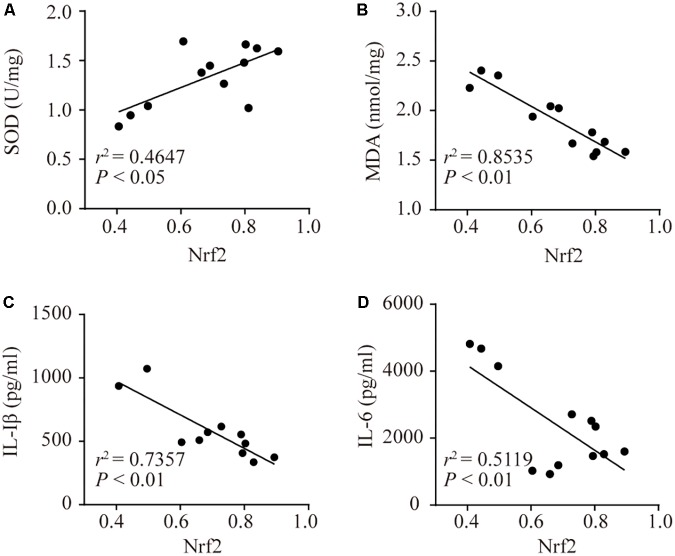
The correlations between Nrf2 and SOD, MDA, IL-1β, and IL-6. Associativity between Nrf2 and SOD (**A**, *r*^2^ = 0.4647, *P <* 0.05), MDA (**B**, *r*^2^ = 0.8535, *P <* 0.01), IL-1β (**C**, *r*^2^ = 0.7357, *P <* 0.01), and IL-6 (**D**, *r*^2^ = 0.5119, *P <* 0.01).

Pearson correlation analyses showed that Nrf2 was positively correlated with SOD (**Figure 6A**). There was a negative correlation between Nrf2 and MDA, IL-1β, and IL-6 (**Figures 6B–D**). This demonstrated that MDA, IL-6, and IL-1β were decreased, but SOD was increased when Nrf2 was activated. This finding implied that activated Nrf2 inhibited oxidative stress and inflammatory responses.

## Discussion

Diabetes mellitus is a common chronic metabolic disease that can cause a variety of complications. As a complication of DM, cognitive dysfunction manifests as impairments in learning and memory, space-time orientation dysfunction, and even in the formation of dementia. Large-scale cross-sectional studies have found that diabetes and dementia are positively correlated (Ahtiluoto et al., 2010). A prospective population-based study found that baseline scores on cognitive function tests in elderly patients with T2DM were lower than those without diabetes (Logroscino et al., 2004). Several clinical studies confirmed that T2DM was associated with cognitive decline (Nooyens et al., 2010; Gao et al., 2016). The diabetes-related cognitive dysfunction appears mild to moderate but can significantly impede daily functioning and seriously decrease the quality of life (Brands et al., 2005). At present, most of the research regarding the diabetes-related cognitive dysfunction is based on STZ-induced diabetic animal models. STZ at low doses partly impairs the function of islet beta cells, resulting in decreased insulin sensitivity in peripheral tissues. A high-fat, high-sugar diet with low doses of STZ can induce T2DM rats (Srinivasan et al., 2005). In this study, we produced T2DM rats using a high-fat, high-sugar diet with STZ intraperitoneally injected at a dose of 35 mg/kg.

Due to the complicated pathogenesis of diabetes-related cognitive dysfunction, there is currently no effective treatment. Therefore, looking for specific and effective drugs for treatment is necessary. AST is a keto carotenoid that is widely found in algae, shrimp, crabs, shellfish, fish and other foods (Higuera-Ciapara et al., 2006). Recent studies have shown that AST elicits the benefits of anti-inflammation, anti-apoptosis, and anti-oxidation, anti-aging, anti-tumor, and immunity enhancement (Wang et al., 2015b). It has been widely applied in the areas of intellectual nourishment, healthcare products, and commercialism (Guerin et al., 2003). The linear portion of the AST molecule contains multiple double bonds, each of which can be either *cis* or *trans*. This enables AST to generate many isomers, of which all-*trans* structures are almost stable and widely disseminated in nature. AST has been proven to exert potent protective effects on diabetic nephropathy in rat models of T2DM by inhibiting expression of oxidative stress and inflammatory mediators (Naito et al., 2004). In addition, it is also effective as an anticoagulant and an anti-inflammatory in diabetic rats (Chan et al., 2012). In T2DM mice, AST ameliorates the apoptosis of retinal ganglion cells by inhibiting oxidative stress (Dong et al., 2013). In the present study, AST alleviated cognitive dysfunction and the structural damage to the hippocampus to some extent, suggesting that AST has a certain therapeutic effect on diabetic cognitive dysfunction.

The role of oxidative stress and inflammatory responses in diabetes-related cognitive dysfunction has gradually attracted the attention of researchers (Zhou et al., 2015; Tian et al., 2016). Under normal and unstressed conditions, the levels of oxidation and of antioxidants are in a relatively balanced state. Under harmful external stress, the oxidation and anti-oxidation systems of an organism lose balance, resulting in the mass production of reactive oxygen species (ROS) and antioxidants that are relatively inadequate (Mittler, 2002). Subsequently, substantial ROS accumulate in the organism, causing lipid peroxidation of polyunsaturated fatty acids to form MDA. Nrf2, a key transcription factor, regulates the expression of cytoprotective and antioxidant genes when it is activated (Nguyen et al., 2009). Under normal conditions, Nrf2 forms a complex with Kelch-like ECH-related protein 1 (Keap1), resulting in the degradation and inactivation of Nrf2 via ubiquitination. When the cell is stimulated by harmful stimuli, Nrf2 separates from Keap1, translocates to the nucleus and binds to the ARE to induce the expression of glutathione-S-transferase, HO-1, NADPH quinine oxidoreductase 1 and other genes (Ma, 2013). This study revealed that AST promotes the expression of Nrf2, HO-1, and SOD, while inhibiting the generation of MDA. From this viewpoint, the protective effects of AST on cognitive dysfunction may be partially due to the recovery of anti-oxidative enzyme levels through Nrf2 activating.

**FIGURE 7:**
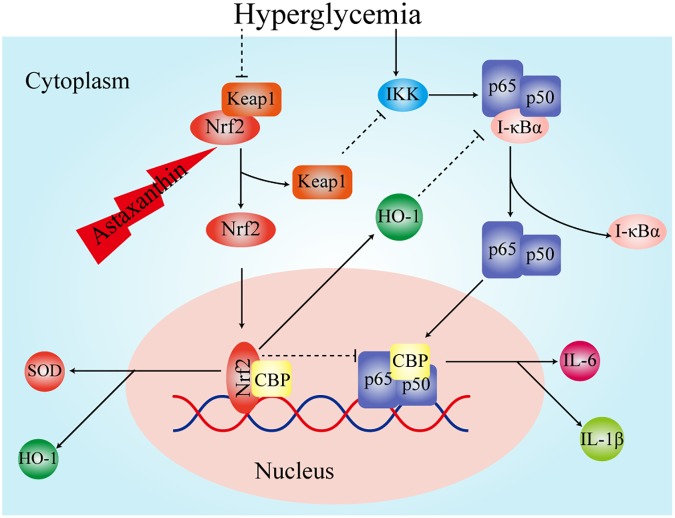
The potential pathway of Nrf2 inhibiting oxidative stress and inflammatory response.

In recent years, a large number of experiments have confirmed that the inflammatory reaction is also closely related to diabetes-related cognitive dysfunction. It has been experimentally confirmed that IL-6, tumor necrosis factor-α, and cyclooxygenase-2 are elevated in the brain tissue of T2DM rats with cognitive dysfunction (Miao et al., 2015). The above studies showed that inflammatory cytokines are involved in the pathogenesis of diabetes-related cognitive dysfunction. In present study we also found the level of pro-inflammatory cytokines IL-1β and IL-6 in chronic diabetic rats were elevated, which was consistent with the opinion that inflammatory cytokines are involved in the pathogenesis of diabetes-related cognitive dysfunction. NF-κB, as one of the regulators of inflammatory responses, plays a key role in the inflammatory response and could promote lipid peroxidation (Menghini et al., 2016; Mitchell et al., 2016). It consists of five protein subunits: RelA (p65), RelB, C-Rel, NF-κB1 (p50 and its precursor p105) and NF-κB2 (p50 and its precursor p105). There is growing evidence that NF-κB is closely linked to synaptic plasticity, memory, and cognitive dysfunction (Kaltschmidt et al., 2006; Ahn et al., 2008). The activation of NF-κB in microglia plays a major role in inflammatory processes. NF-κB is also involved in long-term memory consolidation and fear memory recovery (Boccia et al., 2007). Studies have confirmed that the NF-κB signaling pathway is activated in diabetic rats with cognitive dysfunction (Kuhad et al., 2009). This is consistent with the results of this experiment. At the same time, we found that AST reduced the level of IL-1β and IL-6 in chronic diabetic rats, which implied that AST inhibited the level of inflammatory response. Some experiments confirmed that AST plays a role in lowering blood glucose (Uchiyama et al., 2002), whereas we found that the blood glucose levels were not significantly different between the AST group and the cT2DM group. This might have been due to fact that the treatment time of AST was not long enough. Therefore, the decreased inflammatory responses in the diabetic rats was not caused by hypoglycemia and might be achieved by other pathways. Nrf2 is thought to inhibit the expression of inflammatory cytokines (Ahmed et al., 2017). Previous studies have shown that the expression of inflammatory cytokines is higher in knockout Nrf2 mice than in non-knockout mice (Kong et al., 2015). Experiments have demonstrated that when Nrf2 is activated, Keap1 is isolated from it and then can inhibit the activity of IKK kinase, resulting in NF-κB inactivation (Kim et al., 2010). The carbon monoxide and biliverdin, catalytic products of HO-1, have been shown to suppress the activation of NF-κB (Soares et al., 2004). The transcriptional coactivator, CREB binding protein (CBP), performs its role by activating the transcription process, wherein interaction with transcription factors is managed by its domains (Gerritsen et al., 1997; Wang et al., 2012). The Nrf2 of a free state in the nucleus competitively binds CBP with NF-κB, resulting in impedance of NF-κB activation when the level of Nrf2 increases (Liu et al., 2008; Kim et al., 2013). We surmise that AST improves the expression of Nrf2, and Nrf2 and its intermediate metabolites exert an inhibitory effect on the inflammatory response by inhibiting the activation of NF-κB (**Figure 7**).

In summary, with the assistance of behavioral approaches, morphology, and molecular bioenergetic, this study clarified that AST can effectively alleviate cognitive dysfunction. Although the comprehensive mechanism related to the protective effects of AST on ameliorating the cognitive impairment remains to be fully understood, inhibiting oxidative stress and inflammatory response by the elevation of the Nrf2-ARE signaling may be effective for diabetic cognitive dysfunction.

## Author Contributions

XS and YC contributed conception and design of the study. YF and QL accomplished the experiments. MW and AC performed the statistical analysis. YF and AC wrote the manuscript. XS and YC contributed to English editing. All authors contributed to manuscript revision, read and approved the submitted version.

## Conflict of Interest Statement

The authors declare that the research was conducted in the absence of any commercial or financial relationships that could be construed as a potential conflict of interest.
